# Supplementary material for: Dynamic expectations: Behavioral and electrophysiological evidence of sub-second updates in reward predictions
Source: Commun Biol. 2023 Aug 24;6:871. doi: 10.1038/s42003-023-05199-x (PMC10449862; doi:10.1038/s42003-023-05199-x)
Supplement: Supplementary file 2 — Supplementary Information [file 42003_2023_5199_MOESM2_ESM.pdf]

Supplementary information

Supplementary Note 1: Ratings analyses (EEG Studies 1 and 3)

Participants' raw ratings were converted to a standardized z score, based on each participant's mean and standard deviation for that rating. To test whether participants' happiness and motivation ratings varied as a function of their outcome, we ran mixed model linear regressions with a random effect of outcome, encoded as three dummy variables (Win, Near Win After, Near Win Before; Full Miss serving as a baseline), and a fixed effect of participant. To account for the length of the experiment and the fact that participants reported being less engaged as the game progressed, we ran these regressions separately for the first and the second half of the experiment.

Study 1: Throughout the experiment, participants were happier and more willing to play again after Wins than after all types of misses (Happiness, first half: Win: 1.01 ± 0.10; NWB: -0.16 ± 0.09; NWA: 0.10 ± 0.11; FM: 0.08 ± 0.06; all p's<.001; second Half: Win: 0.64 ± 0.10; NWB: -0.35 ± 0.09; NWA: -0.37 ± 0.08; FM: -0.32 ± 0.05, all p's<.001; Motivation, first half: Win: 0.90 ± 0.11; NWB: .25 ± 0.10; NWA: 0.44 ± 0.11; FM: 0.32 ± 0.06; all p's<=.001; second half: (Win: 0.02 ± 0.10; NWB: -0.46 ± 0.08; NWA: -0.51 ± 0.08; FM: -0.45 ± 0.05, all p's<.001). In addition, in the first half of the experiment, participants were less happy following NWB compared to NWA (p=0.051) and FM (p=0.025), and more willing to play again after NWA than NWB (p=.096). There were no differences between the different types of misses in the second half (all p's>0.7). These results are in line with previous studies showing that Near Win (Before and After pooled together) are less pleasant than Full Misses but increase willingness to play again[1–4]. The few studies separating NWB from NWA report conflicting effects, with some studies showing that these two effects are mostly driven by Near Win After[2,5], and others showing different effects of NWB and NWA[6]. The fact these Near Wins effects disappeared in the second half of the experiment suggests that they are temporally limited, or that participants got bored or tired of the game at some point.

Study 3: The behavioral results were similar to those of Study 1. Throughout the experiment, participants were less happy and less willing to play again after Losses than after all types of Escapes (Happiness, first half: Loss: -0.67 ± 0.11; NLB: 0.68 ± 0.10; NLA: 0.21 ± 0.11; FE: 0.25 ± 0.06; all p's<.001; second Half: Loss: -.98 ± 0.07; NLB: -0.10 ± 0.09; NLA: -0.01 ± 0.08; FE: 0.02 ± 0.05, all p's<.001; Motivation, first half: Loss: -0.10 ± 0.13; NLB: .77 ± 0.11; NLA: 0.39 ± 0.09; FE: 0.41 ± 0.05; all p's<=.001; second half: Loss: -0.76 ± 0.10; NLB: -0.34 ± 0.08; NLA: -0.26 ± 0.09; FE: -0.27 ± 0.05, all p's<.001). In addition, in the first half of the experiment, participants were happier and more willing to play again following NLB compared to NLA and FE (p's<.003). There were no differences in the second half (p's>0.229).

Supplementary Note 2: Association of behavioral expectation trajectories with EEG responses with a lag

In the article, we presented an analysis examining whether single subjects' EEG data during the deceleration phase correlate with the behavioral expectations trajectories. In the original model, we only had one variable - the beginning of the time-window used to calculate the correlation between the two timeseries (see Methods). However, one of the two timeseries might precede the other, and allowing for a lag between the timeseries could increase their correlation. Here we present the results of a model with lag as a second variable parameter.

The lag results are very similar to the ones we obtained without the lag. For Studies 1 and 2 (regular slot machine), we found an absolute correlation of 0.45 ($p<.001$) (vs. 0.44, $p<.001$ without lag) between the two timeseries for a time-window running from -0.97 to 0 seconds for the EEG data and from -0.9 to 0.07 seconds for behavior. For Studies 3 and 4 (opposite slot machine), we found an absolute correlation of 0.39 ($p<.001$) (vs. 0.39, $p<.001$ without lag) for a time-window running from -1.27 to 0 seconds for the EEG data and from -1.17 to 0.08 seconds for behavior. In both cases, the best correlation was obtained when the behavioral timeseries preceded the EEG by 0.07-0.08 seconds.

We had no clear a priori hypothesis regarding the direction or the value of the lag. On one hand, one could expect the EEG to precede behavior, as EEG tracks cognitive processes with millisecond precision, whereas behavioral tasks require a decision and a button press, which is bound to introduce a delay. On the other hand, it is important to remember that participants did not play the same games in the EEG and behavioral sessions. Participants playing the EEG task have been less engaged and less attentive than participants in the Slot or Not behavioral task. Indeed, in the EEG task, participants were obligated to bet on the slot machine, and once they chose an item, they were passive viewers of the reel spinning. In the behavioral task, participants could make betting decisions during the spinning and deceleration phases, making the game more engaging, and potentially increasing attention to the deceleration phase.

Supplementary Note 3: Association of behavioral expectation trajectories with EEG responses with a lag

In the article, we presented an analysis examining whether single subjects' EEG data during the deceleration phase correlate with the behavioral expectations trajectories. In the original model, we only had one variable - the beginning of the time-window used to calculate the correlation between the two timeseries (see Methods). However, one of the two timeseries might precede the other, and allowing for a lag between the timeseries could increase their correlation. Here we present the results of a model with lag as a second variable parameter.

The lag results are very similar to the ones we obtained without the lag. For Studies 1 and 2 (regular slot machine), we found an absolute correlation of 0.45 ($p<.001$) (vs. 0.44, $p<.001$ without

lag) between the two timeseries for a time-window running from -0.97 to 0 seconds for the EEG data and from -0.9 to 0.07 seconds for behavior. For Studies 3 and 4 (opposite slot machine), we found an absolute correlation of 0.39 ($p<.001$) (vs. 0.39, $p<.001$ without lag) for a time-window running from -1.27 to 0 seconds for the EEG data and from -1.17 to 0.08 seconds for behavior. In both cases, the best correlation was obtained when the behavioral timeseries preceded the EEG by 0.07-0.08 seconds.

We had no clear a priori hypothesis regarding the direction or the value of the lag. On one hand, one could expect the EEG to precede behavior, as EEG tracks cognitive processes with millisecond precision, whereas behavioral tasks require a decision and a button press, which is bound to introduce a delay. On the other hand, it is important to remember that participants did not play the same games in the EEG and behavioral sessions. Participants playing the EEG task have been less engaged and less attentive than participants in the Slot or Not behavioral task. Indeed, in the EEG task, participants were obligated to bet on the slot machine, and once they chose an item, they were passive viewers of the reel spinning. In the behavioral task, participants could make betting decisions during the spinning and deceleration phases, making the game more engaging, and potentially increasing attention to the deceleration phase.

Supplementary Table 1: Study 1's EEG Repeated-Measure ANOVA results [-3000 -2500ms]

(Note that Tukey pairwise comparisons are presented for Repeated-Measure ANOVAs that reached significance ($p<.008$)).

			Prob > F			
Source	df	F	Regular	H-F	G-G	Box
outcome	3	0.89	0.4475	0.4369	0.4306	0.3512
Residual	105					

Supplementary Table 2: Study 1's EEG Repeated-Measure ANOVA results [-2500 -2000ms]

			Prob > F			
Source	df	F	Regular	H-F	G-G	Box
outcome	3	1.93	0.129	0.144	0.1477	0.1734
Residual	105					

Supplementary Table 3: Study 1's EEG Repeated-Measure ANOVA results [-2000 -1500ms]

			Prob > F			
Source	df	F	Regular	H-F	G-G	Box
outcome	3	5.72	0.0012	0.0023	0.003	0.0223
Residual	105					

			Tukey		Tukey	
	Contrast	Std. err.	t	P>t	[95% conf.	interval]
NWB vs Win	2.091598	0.735839	2.84	0.027	0.170576	4.012619
NWA vs Win	-0.84219	0.735839	-1.14	0.663	-2.76322	1.078827
FM vs Win	0.086329	0.735839	0.12	0.999	-1.83469	2.00735
NWA vs NWB	-2.93379	0.735839	-3.99	0.001	-4.85481	-1.01277
FM vs NWB	-2.00527	0.735839	-2.73	0.037	-3.92629	-0.08425
FM vs NWA	0.928523	0.735839	1.26	0.589	-0.9925	2.849544

Supplementary Table 4: Study 1's EEG Repeated-Measure ANOVA results [-1500 -1000ms]

			Prob>F			
Source	df	F	Regular	H-F	G-G	Box
outcome	3	14.87	<.001	<.001	<.001	0.0005
Residual	105					

			Tukey		Tukey	
	Contrast	Std. err.	t	P>t	[95% conf.	interval]
NWB vs Win	3.682712	0.734276	5.02	<.001	1.765773	5.599652
NWA vs Win	-0.56037	0.734276	-0.76	0.871	-2.47731	1.356574
FM vs Win	2.406731	0.734276	3.28	0.008	0.489791	4.32367
NWA vs NWB	-4.24308	0.734276	-5.78	<.001	-6.16002	-2.32614
FM vs NWB	-1.27598	0.734276	-1.74	0.31	-3.19292	0.640958
FM vs NWA	2.967096	0.734276	4.04	0.001	1.050156	4.884036

Supplementary Table 5: Study 1's EEG Repeated-Measure ANOVA results [-1000 -500ms]

			Prob > F			
Source	df	F	Regular	H-F	G-G	Box
outcome	3	27.5	<.001	<.001	<.001	<.001
Residual	105					

			Tukey		Tukey	
	Contrast	Std. err.	t	P>t	[95% conf.	interval]
NWB vs Win	4.74195	0.797405	5.95	<.001	2.6602	6.823699
NWA vs Win	-0.04877	0.797405	-0.06	1	-2.13052	2.032978
FM vs Win	5.408381	0.797405	6.78	<.001	3.326631	7.49013
NWA vs NWB	-4.79072	0.797405	-6.01	<.001	-6.87247	-2.70897
FM vs NWB	0.666431	0.797405	0.84	0.837	-1.41532	2.748181
FM vs NWA	5.457152	0.797405	6.84	<.001	3.375403	7.538902

Supplementary Table 6: Study 1's EEG Repeated-Measure ANOVA results [-500 0ms]

			Prob > F			
Source	df	F	Regular	H-F	G-G	Box
outcome	3	13.37	<.001	<.001	<.001	0.0008
Residual	105					

			Tukey		Tukey	
	Contrast	Std. err.	t	P>t	[95% conf.	interval]
NWB vs Win	1.044936	0.813267	1.28	0.575	-1.07822	3.168096
NWA vs Win	4.467786	0.813267	5.49	<.001	2.344626	6.590945
FM vs Win	3.605879	0.813267	4.43	<.001	1.482719	5.729039
NWA vs NWB	3.42285	0.813267	4.21	<.001	1.29969	5.546009
FM vs NWB	2.560943	0.813267	3.15	0.011	0.437783	4.684103
FM vs NWA	-0.86191	0.813267	-1.06	0.715	-2.98507	1.261253

Supplementary Table 7: Study 1's EEG Repeated-Measure ANOVA results - FRN

			Prob > F			
Source	df	F	Regular	H-F	G-G	Box
outcome	3	29.31	<.001	<.001	<.001	<.001
Residual	105					

	Contrast	Std. err.	Tukey t	P>t	Tukey [95% conf.	interval]
NWB vs Win	-7.80911	0.931541	-8.38	<.001	-10.241	-5.37718
NWA vs Win	-6.84022	0.931541	-7.34	<.001	-9.27215	-4.40829
FM vs Win	-6.48293	0.931541	-6.96	<.001	-8.91487	-4.051
NWA vs NWB	0.968893	0.931541	1.04	0.726	-1.46304	3.400824
FM vs NWB	1.326178	0.931541	1.42	0.488	-1.10575	3.758109
FM vs NWA	0.357285	0.931541	0.38	0.981	-2.07465	2.789216

Supplementary Table 8: Study 1's EEG Repeated-Measure ANOVA results – P3

			Prob > F			
Source	df	F	Regular	H-F	G-G	Box
outcome	3	42.57	<.001	<.001	<.001	<.001
Residual	105					

	Contrast	Std. err.	Tukey t	P>t	Tukey [95% conf.	interval]
NWB vs Win	-4.51423	0.867508	-5.2	<.001	-6.779	-2.24947
NWA vs Win	-8.83128	0.867508	-10.18	<.001	-11.096	-6.56652
FM vs Win	-7.92357	0.867508	-9.13	<.001	-10.1883	-5.65881
NWA vs NWB	-4.31705	0.867508	-4.98	<.001	-6.58181	-2.05229
FM vs NWB	-3.40934	0.867508	-3.93	0.001	-5.67411	-1.14458
FM vs NWA	0.907706	0.867508	1.05	0.723	-1.35706	3.17247

Supplementary Table 9: Study 2 "Slot or Not"'s Repeated-Measure ANOVA [-3000 -2500ms]

			Prob > F			
Source	df	F	Regular	H-F	G-G	Box
outcome	3	0.21	0.8885	0.8696	0.8518	0.6493
Residual	87					

Supplementary Table 10: Study 2 "Slot or Not"'s Repeated-Measure ANOVA [-2500 -2000ms]

			Prob > F			
Source	df	F	Regular	H-F	G-G	Box
outcome	3	0.49	0.6874	0.6574	0.641	0.4878
Residual	87					

Supplementary Table 11: Study 2 "Slot or Not"'s Repeated-Measure ANOVA [-2000 -1500ms]

			Prob > F			
Source	df	F	Regular	H-F	G-G	Box
outcome	3	1.61	0.1923	0.1967	0.2008	0.2142
Residual	87					

Supplementary Table 12: Study 2 "Slot or Not"'s Repeated-Measure ANOVA [-1500 -1000ms]

			Prob > F			
Source	df	F	Regular	H-F	G-G	Box
outcome	3	11.34	<.001	<.001	<.001	0.0022
Residual	87					

			Tukey		Tukey	
	Contrast	Std. err.	t	P>t	[95% conf.	interval]
NWB vs Win	-0.06833	0.03848	-1.78	0.292	-0.16913	0.032461
NWA vs Win	0.144444	0.03848	3.75	0.002	0.04365	0.245239
FM vs Win	-0.02056	0.03848	-0.53	0.95	-0.12135	0.080239
NWA vs NWB	0.212778	0.03848	5.53	<.001	0.111984	0.313572
FM vs NWB	0.047778	0.03848	1.24	0.602	-0.05302	0.148572
FM vs NWA	-0.165	0.03848	-4.29	<.001	-0.26579	-0.06421

Supplementary Table 13: Study 2 "Slot or Not"'s Repeated-Measure ANOVA [-1000 -500ms]

			Prob > F			
Source	df	F	Regular	H-F	G-G	Box
outcome	3	33.63	<.001	<.001	<.001	<.001
Residual	87					

			Tukey		Tukey	
	Contrast	Std. err.	t	P>t	[95% conf.	interval]
NWB vs Win	-0.17056	0.033	-5.17	<.001	-0.257	-0.08412
NWA vs Win	0.096111	0.033	2.91	0.023	0.009671	0.182552
FM vs Win	-0.18167	0.033	-5.51	<.001	-0.26811	-0.09523
NWA vs NWB	0.266667	0.033	8.08	<.001	0.180226	0.353107
FM vs NWB	-0.01111	0.033	-0.34	0.987	-0.09755	0.075329
FM vs NWA	-0.27778	0.033	-8.42	<.001	-0.36422	-0.19134

Supplementary Table 14: Study 2 "Slot or Not"'s Repeated-Measure ANOVA [-500 0ms]

			Prob > F			
Source	df	F	Regular	H-F	G-G	Box
outcome	3	19.06	<.001	<.001	<.001	0.0001
Residual	87					

			Tukey		Tukey	
	Contrast	Std. err.	t	P>t	[95% conf.	interval]
2 vs 1	-0.23278	0.048683	-4.78	<.001	-0.3603	-0.10526
3 vs 1	-0.205	0.048683	-4.21	<.001	-0.33252	-0.07748
4 vs 1	-0.36333	0.048683	-7.46	<.001	-0.49085	-0.23581
3 vs 2	0.027778	0.048683	0.57	0.941	-0.09974	0.155299
4 vs 2	-0.13056	0.048683	-2.68	0.043	-0.25808	-0.00303
4 vs 3	-0.15833	0.048683	-3.25	0.009	-0.28585	-0.03081

Supplementary Table 15: Study 3's EEG Repeated-Measure ANOVA results [-3000 -2500ms]

			Prob > F			
Source	Df	F	Regular	H-F	G-G	Box
outcome	3	1.43	0.2371	0.2397	0.2417	0.2393
Residual	102					

Supplementary Table 16: Study 3's EEG Repeated-Measure ANOVA results [-2500 -2000ms]

			Prob > F			
Source	Df	F	Regular	H-F	G-G	Box
outcome	3	3.53	0.0175	0.0246	0.0279	0.0688
Residual	102					

Supplementary Table 17: Study 3's EEG Repeated-Measure ANOVA results [-2000 -1500ms]

			Prob > F			
Source	Df	F	Regular	H-F	G-G	Box
outcome	3	7.48	<.001	<.001	<.001	0.0098
Residual	102					

			Tukey		Tukey	
	Contrast	Std. err.	t	P>t	[95% conf.	interval]
NLB vs Loss	0.94813	0.720067	1.32	0.554	-0.93261	2.828867
NLA vs Loss	-1.61891	0.720067	-2.25	0.117	-3.49965	0.261823
FE vs Loss	-2.03004	0.720067	-2.82	0.029	-3.91077	-0.1493
NLA vs NLB	-2.56704	0.720067	-3.57	0.003	-4.44778	-0.68631
FE vs NLB	-2.97817	0.720067	-4.14	<.001	-4.8589	-1.09743
FE vs NLA	-0.41112	0.720067	-0.57	0.941	-2.29186	1.469614

Supplementary Table 18: Study 3's EEG Repeated-Measure ANOVA results [-1500 -1000ms]

			Prob>F			
Source	Df	F	Regular	H-F	G-G	Box
outcome	3	9.31	<.001	<.001	0.0001	0.0044
Residual	102					
			Tukey		Tukey	
	Contrast	Std. err.	t	P>t	[95% conf.	interval]
NLB vs Loss	1.305904	0.707803	1.85	0.259	-0.5428	3.154611
NLA vs Loss	-2.31367	0.707803	-3.27	0.008	-4.16237	-0.46496
FE vs Loss	-0.94363	0.707803	-1.33	0.544	-2.79234	0.905077
NLA vs NLB	-3.61957	0.707803	-5.11	<.001	-5.46828	-1.77087
FE vs NLB	-2.24953	0.707803	-3.18	0.01	-4.09824	-0.40083
FE vs NLA	1.370038	0.707803	1.94	0.22	-0.47867	3.218745

Supplementary Table 19: Study 3's EEG Repeated-Measure ANOVA results [-1000 -500ms]

			Prob > F			
Source	Df	F	Regular	H-F	G-G	Box
outcome	3	17.08	<.001	<.001	<.001	0.0002
Residual	102					

			Tukey		Tukey	
	Contrast	Std. err.	t	P>t	[95% conf.	interval]
NLB vs Loss	2.596125	0.807639	3.21	0.009	0.486659	4.70559
NLA vs Loss	-2.44756	0.807639	-3.03	0.016	-4.55703	-0.3381
FE vs Loss	2.356701	0.807639	2.92	0.022	0.247235	4.466166
NLA vs NLB	-5.04369	0.807639	-6.24	<.001	-7.15315	-2.93422
FE vs NLB	-0.23942	0.807639	-0.3	0.991	-2.34889	1.870041
FE vs NLA	4.804262	0.807639	5.95	<.001	2.694796	6.913727

Supplementary Table 20: Study 3's EEG Repeated-Measure ANOVA results [-500 0ms]

			Prob > F			
Source	Df	F	Regular	H-F	G-G	Box
outcome	3	8.83	<.001	<.001	0.0001	0.0054
Residual	102					

			Tukey		Tukey	
	Contrast	Std. err.	t	P>t	[95% conf.	interval]
NLB vs Loss	0.198744	0.829179	0.24	0.995	-1.96698	2.364472
NLA vs Loss	3.32327	0.829179	4.01	0.001	1.157542	5.488998
FE vs Loss	2.870044	0.829179	3.46	0.004	0.704316	5.035772
NLA vs NLB	3.124526	0.829179	3.77	0.002	0.958798	5.290255
FE vs NLB	2.671301	0.829179	3.22	0.009	0.505572	4.837029
FE vs NLA	-0.45323	0.829179	-0.55	0.947	-2.61895	1.712502

Supplementary Table 21: Study 3's EEG Repeated-Measure ANOVA results - FRN

			Prob > F			
Source	df	F	Regular	H-F	G-G	Box
outcome	3	26.22	<.001	<.001	<.001	<.001
Residual	102					

			Tukey		Tukey	
	Contrast	Std. err.	t	P>t	[95% conf.	interval]
NLB vs Loss	5.869042	0.952402	6.16	<.001	3.381469	8.356615
NLA vs Loss	7.712022	0.952402	8.1	<.001	5.224449	10.19959
FE vs Loss	6.604507	0.952402	6.93	<.001	4.116934	9.09208
NLA vs NLB	1.84298	0.952402	1.94	0.22	-0.64459	4.330553
FE vs NLB	0.735465	0.952402	0.77	0.867	-1.75211	3.223038
FE vs NLA	-1.10752	0.952402	-1.16	0.652	-3.59509	1.380058

Supplementary Table 22: Study 3's EEG Repeated-Measure ANOVA results – P3

			Prob > F			
Source	df	F	Regular	H-F	G-G	Box
outcome	3	31.52	<.001	<.001	<.001	<.001
Residual	102					

			Tukey		Tukey	
	Contrast	Std. err.	t	P>t	[95% conf.	interval]
NLB vs Loss	-1.49612	1.053267	-1.42	0.49	-4.24714	1.254904
NLA vs Loss	-8.29418	1.053267	-7.87	<.001	-11.0452	-5.54316
FE vs Loss	-7.48593	1.053267	-7.11	<.001	-10.237	-4.73491
NLA vs NLB	-6.79807	1.053267	-6.45	<.001	-9.54909	-4.04705
FE vs NLB	-5.98982	1.053267	-5.69	<.001	-8.74084	-3.2388
FE vs NLA	0.80825	1.053267	0.77	0.869	-1.94277	3.55927

Supplementary Table 23: Study 4 "Slot or Not"'s Repeated-Measure ANOVA [-3000 -2500ms]

			Prob > F			
Source	df	F	Regular	H-F	G-G	Box
outcome	3	0.02	0.9972	0.9954	0.9917	0.9006
Residual	60					

Supplementary Table 24: Study 4 “Slot or Not”’s Repeated-Measure ANOVA [-2500 -2000ms]

			Prob > F			
Source	df	F	Regular	H-F	G-G	Box
outcome	3	0.14	0.9363	0.8751	0.8568	0.7131
Residual	60					

Supplementary Table 25: Study 4 “Slot or Not”’s Repeated-Measure ANOVA [-2000 -1500ms]

			Prob > F			
Source	df	F	Regular	H-F	G-G	Box
outcome	3	1.21	0.3126	0.3089	0.3069	0.2837
Residual	60					

Supplementary Table 26: Study 4 “Slot or Not”’s Repeated-Measure ANOVA [-1500 -1000ms]

			Prob > F			
Source	df	F	Regular	H-F	G-G	Box
outcome	3	6.68	0.0006	0.0015	0.0023	0.0177
Residual	60					

			Tukey		Tukey	
	Contrast	Std. err.	t	P>t	[95% conf.	interval]
NLB vs Loss	-0.00476	0.061671	-0.08	1	-0.16773	0.158206
NLA vs Loss	-0.21429	0.061671	-3.47	0.005	-0.37725	-0.05132
FE vs Loss	0.031217	0.061671	0.51	0.957	-0.13175	0.194185
NLA vs NLB	-0.20952	0.061671	-3.4	0.006	-0.37249	-0.04656
FE vs NLB	0.035979	0.061671	0.58	0.937	-0.12699	0.198946
FE vs NLA	0.245503	0.061671	3.98	0.001	0.082535	0.40847

Supplementary Table 27: Study 4 "Slot or Not"'s Repeated-Measure ANOVA [-1000 -500ms]

			Prob > F			
Source	df	F	Regular	H-F	G-G	Box
outcome	3	10.99	0	0	0.0001	0.0035
Residual	60					
			Tukey		Tukey	
	Contrast	Std. err.	t	P>t	[95% conf.	interval]
NLB vs Loss	0.144444	0.067318	2.15	0.151	-0.03344	0.322333
NLA vs Loss	-0.10794	0.067318	-1.6	0.385	-0.28582	0.069952
FE vs Loss	0.250529	0.067318	3.72	0.002	0.072641	0.428417
NLA vs NLB	-0.25238	0.067318	-3.75	0.002	-0.43027	-0.07449
FE vs NLB	0.106085	0.067318	1.58	0.4	-0.0718	0.283973
FE vs NLA	0.358466	0.067318	5.32	<.001	0.180577	0.536354

Supplementary Table 28: Study 4 "Slot or Not"'s Repeated-Measure ANOVA [-500 0ms]

			Prob > F			
Source	df	F	Regular	H-F	G-G	Box
outcome	3	11.23	0	0	0.0001	0.0032
Residual	60					

			Tukey		Tukey	
	Contrast	Std. err.	t	P>t	[95% conf.	interval]
NLB vs Loss	0.206349	0.071026	2.91	0.026	0.018662	0.394036
NLA vs Loss	0.184127	0.071026	2.59	0.056	-0.00356	0.371814
FE vs Loss	0.411376	0.071026	5.79	<.001	0.223689	0.599063
NLA vs NLB	-0.02222	0.071026	-0.31	0.989	-0.20991	0.165465
FE vs NLB	0.205026	0.071026	2.89	0.027	0.01734	0.392713
FE vs NLA	0.227249	0.071026	3.2	0.012	0.039562	0.414936

Supplementary Figure 1 – Study 1's FRN and P3

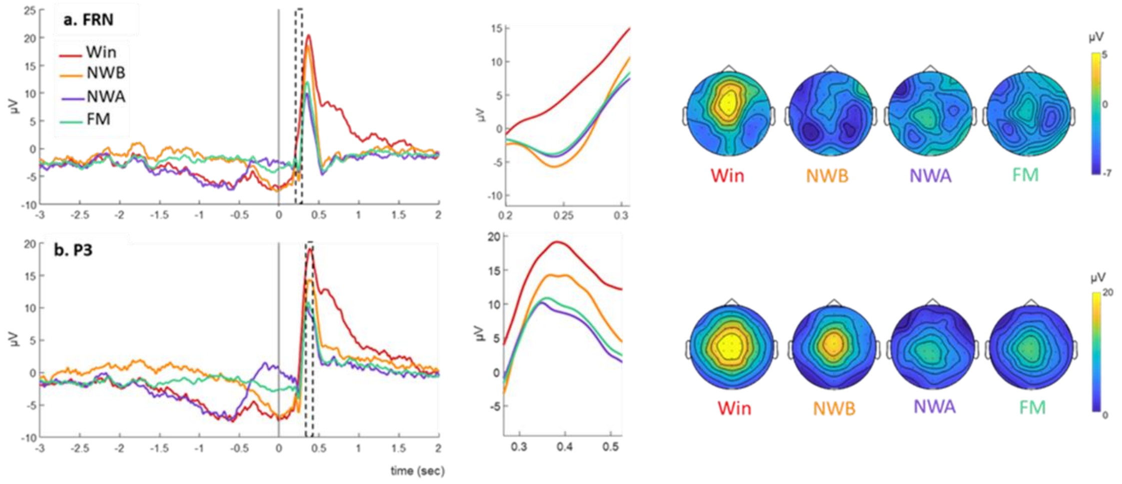

a. FRN results: Left panel shows the grand average ERPs for each outcome locked to the stop of the machine at FRN electrodes of interest pooled (Fz, FCz, Cz). The dashed rectangle over the waveforms indicates the time window used for the FRN analysis. Middle panel shows a zoom in of that time window. Right panel shows the topographies in µV for each outcome. **b. P3 results:** Same for P3, for average of electrodes Cz, CPz, Pz, POz, Oz.

Supplementary Fig. 2 – Study 3's FRN and P3

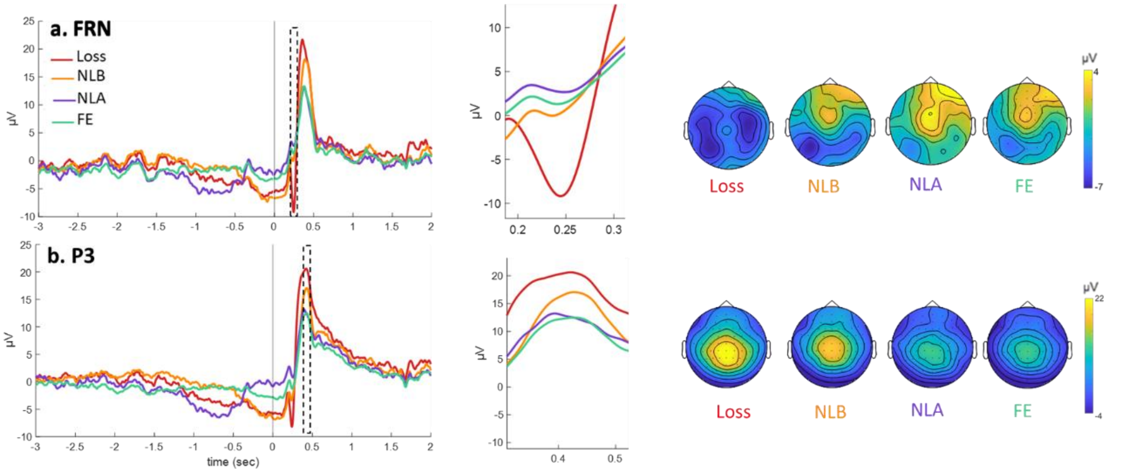

a. FRN results: Left panel shows the grand average ERPs for each outcome locked to the stop of the machine at FRN electrodes of interest pooled (Fz, FCz, Cz). The dashed rectangle over the waveforms indicates the time window used for the FRN analysis. Middle panel shows a zoom in of that time window. Right panel shows the topographies in µV for each outcome. **b. P3 results:** Same for P3, for average of electrodes Cz, CPz, Pz, POz, Oz.

Supplementary Figure 3 – Difference topographies during the last second of deceleration for Studies 1 and 3

a. Study 1

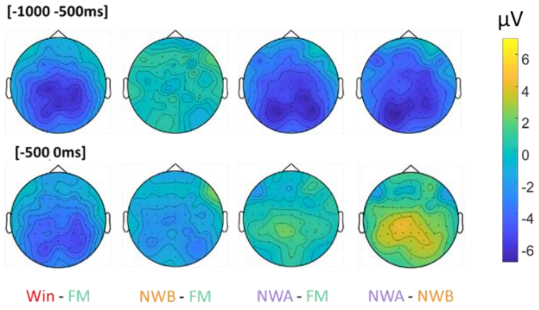

b. Study 3

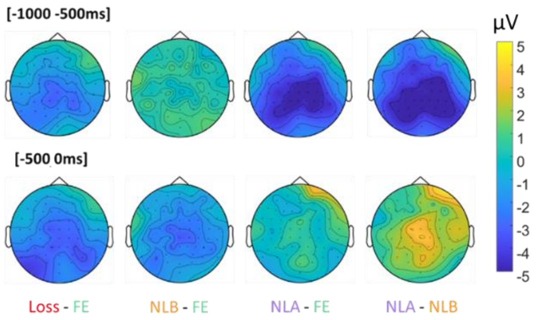

a. Difference topographies for Study 1 during the last second of deceleration. Upper topographies: [-1000 -500ms], lower topographies: [-500 0ms]). Each topography represents the difference in µV between the topographies for the two outcomes indicated at the bottom. From left to right: Win minus Full Miss, Near Win Before minus Full Miss, Near Win After minus Full Miss, Near Win After minus Near Win Before. **b. Same for Study 3.** From left to right: Loss minus Full Escape, Near Loss Before minus Full Escape, Near Loss After minus Full Escape, Near Loss After minus Near Loss Before.

Supplementary references

1. Billieux, J., Van der Linden, M., Khazaal, Y., Zullino, D. & Clark, L. Trait gambling cognitions predict near-miss experiences and persistence in laboratory slot machine gambling. *British Journal of Psychology* **103**, 412–427 (2012).

2. Clark, L., Lawrence, A. J., Astley-Jones, F. & Gray, N. Gambling Near-Misses Enhance Motivation to Gamble and Recruit Win-Related Brain Circuitry. *Neuron* **61**, 481–490 (2009).

3. Cote, D., Caron, A., Aubert, J., Desrochers, V. & Ladouceur, R. Near Wins Prolong Gambling on a Video Lottery Terminal. *JOURNAL OF GAMBLING STUDIES* (2003).

4. Qi, S., Ding, C., Song, Y. & Yang, D. Neural correlates of near-misses effect in gambling. *Neuroscience Letters* **493**, 80–85 (2011).
5. Wu, Y., van Dijk, E., Li, H., Aitken, M. & Clark, L. On the Counterfactual Nature of Gambling Near-misses: An Experimental Study. *Journal of Behavioral Decision Making* **30**, 855–868 (2017).
6. Sharman, S., Aitken, M. R. & Clark, L. Dual effects of 'losses disguised as wins' and near-misses in a slot machine game. *International Gambling Studies* **15**, 212–223 (2015).
7. Gehring, W. J. & Willoughby, A. R. The Medial Frontal Cortex and the Rapid Processing of Monetary Gains and Losses. *Science* **295**, 2279–2282 (2002).
8. Hajcak, G., Moser, J. S., Holroyd, C. B. & Simons, R. F. The feedback-related negativity reflects the binary evaluation of good versus bad outcomes. *Biological Psychology* **71**, 148–154 (2006).
9. Lole, L., Gonsalvez, C. J., Barry, R. J. & De Blasio, F. M. Can event-related potentials serve as neural markers for wins, losses, and near-wins in a gambling task? A principal components analysis. *International Journal of Psychophysiology* **89**, 390–398 (2013).
10. Lole, L., Gonsalvez, C. J. & Barry, R. J. Reward and punishment hyposensitivity in problem gamblers: A study of event-related potentials using a principal components analysis. *Clinical Neurophysiology* **126**, 1295–1309 (2015).
11. Luo, Q., Wang, Y. & Qu, C. The near-miss effect in slot-machine gambling: modulation of feedback-related negativity by subjective value. *NeuroReport* **22**, 989 (2011).
12. Bellebaum, C., Polezzi, D. & Daum, I. It is less than you expected: The feedback-related negativity reflects violations of reward magnitude expectations. *Neuropsychologia* **48**, 3343–3350 (2010).

13. Bismark, A. W., Hajcak, G., Whitworth, N. M. & Allen, J. J. B. The role of outcome expectations in the generation of the feedback-related negativity. *Psychophysiology* **50**, 125–133 (2013).

14. Cohen, M. X., Elger, C. E. & Ranganath, C. Reward expectation modulates feedback-related negativity and EEG spectra. *NeuroImage* **35**, 968–978 (2007).

15. Hajcak, G., Moser, J. S., Holroyd, C. B. & Simons, R. F. It's worse than you thought: The feedback negativity and violations of reward prediction in gambling tasks. *Psychophysiology* **44**, 905–912 (2007).

16. Marciano, D., Bentin, S. & Deouell, L. Y. Alternative outcomes create biased expectations regarding the received outcome: Evidence from event-related potentials. *Neuropsychologia* **113**, 126–139 (2018).

17. Yu, R. & Zhou, X. To Bet or Not to Bet? The Error Negativity or Error-related Negativity Associated with Risk-taking Choices. *Journal of Cognitive Neuroscience* **21**, 684–696 (2009).

18. Zhang, Y., Li, X., Qian, X. & Zhou, X. Brain responses in evaluating feedback stimuli with a social dimension. *Front. Hum. Neurosci.* **6**, (2012).

19. Hajcak, G., Holroyd, C. B., Moser, J. S. & Simons, R. F. Brain potentials associated with expected and unexpected good and bad outcomes. *Psychophysiology* **42**, 161–170 (2005).

20. Hoy, C. W., Steiner, S. C. & Knight, R. T. Single-trial modeling separates multiple overlapping prediction errors during reward processing in human EEG. *Commun Biol* **4**, 1–17 (2021).

21. Wu, Y. & Zhou, X. The P300 and reward valence, magnitude, and expectancy in outcome evaluation. *Brain Research* **1286**, 114–122 (2009).

22. Dores, A. R. *et al.* Neurophysiological Correlates of the Near-Miss Effect in Gambling. *J Gambl Stud* **36**, 653–668 (2020).

23. Philiastides, M. G., Biele, G., Vavatzanidis, N., Kazzer, P. & Heekeren, H. R. Temporal dynamics of prediction error processing during reward-based decision making. *NeuroImage* **53**, 221–232 (2010).

24. Yeung, N. Independent Coding of Reward Magnitude and Valence in the Human Brain. *Journal of Neuroscience* **24**, 6258–6264 (2004).

25. Alicart, H., Cucurell, D., Mas-Herrero, E. & Marco-Pallarés, J. Human oscillatory activity in near-miss events. *Social Cognitive and Affective Neuroscience* **10**, 1405–1412 (2015).

26. Ulrich, N. & Hewig, J. A miss is as good as a mile? Processing of near and full outcomes in a gambling paradigm. *Psychophysiology* **51**, 819–823 (2014).

27. Li, P. *et al.* The influence of the diffusion of responsibility effect on outcome evaluations: Electrophysiological evidence from an ERP study. *NeuroImage* **52**, 1727–1733 (2010).

28. Yeung, N., Holroyd Clay B., & Cohen, Jonathan D. ERP Correlates of Feedback and Reward Processing in the Presence and Absence of Response Choice | Cerebral Cortex | Oxford Academic. **15**, 535–544 (2005).

29. Sato, A. *et al.* Effects of value and reward magnitude on feedback negativity and P300. *NeuroReport* **16**, 407 (2005).
