# Supplementary material for: Dynamic expectations: Behavioral and electrophysiological evidence of sub-second updates in reward predictions
Source: Commun Biol. 2023 Aug 24;6:871. doi: 10.1038/s42003-023-05199-x (PMC10449862; doi:10.1038/s42003-023-05199-x)
Supplement: Supplementary file 5 — Reporting summary [file 42003_2023_5199_MOESM5_ESM.pdf]

nature portfolio

Corresponding author(s): Deborah Marciano

Last updated by author(s): Jun 20, 2023

Reporting Summary

Nature Portfolio wishes to improve the reproducibility of the work that we publish. This form provides structure for consistency and transparency in reporting. For further information on Nature Portfolio policies, see our Editorial Policies and the Editorial Policy Checklist.

Statistics

For all statistical analyses, confirm that the following items are present in the figure legend, table legend, main text, or Methods section.

n/a	Confirmed	
☐	☒	The exact sample size (*n*) for each experimental group/condition, given as a discrete number and unit of measurement
☐	☒	A statement on whether measurements were taken from distinct samples or whether the same sample was measured repeatedly
☐	☒	The statistical test(s) used AND whether they are one- or two-sided<br>*Only common tests should be described solely by name; describe more complex techniques in the Methods section.*
☐	☒	A description of all covariates tested
☐	☒	A description of any assumptions or corrections, such as tests of normality and adjustment for multiple comparisons
☐	☒	A full description of the statistical parameters including central tendency (e.g. means) or other basic estimates (e.g. regression coefficient) AND variation (e.g. standard deviation) or associated estimates of uncertainty (e.g. confidence intervals)
☐	☒	For null hypothesis testing, the test statistic (e.g. *F*, *t*, *r*) with confidence intervals, effect sizes, degrees of freedom and *P* value noted<br>*Give P values as exact values whenever suitable.*
☒	☐	For Bayesian analysis, information on the choice of priors and Markov chain Monte Carlo settings
☐	☒	For hierarchical and complex designs, identification of the appropriate level for tests and full reporting of outcomes
☐	☒	Estimates of effect sizes (e.g. Cohen's *d*, Pearson's *r*), indicating how they were calculated

Our web collection on statistics for biologists contains articles on many of the points above.

Software and code

Policy information about availability of computer code

Data collection	Neurobehavioral Systems Presentation (version 14.1)
Data analysis	Matlab + Fieldtrip Toolbox<br>Stata 17<br><br>Original code was deposited on Zenodo and is available for public download (https://doi.org/10.5281/zenodo.8048382).

For manuscripts utilizing custom algorithms or software that are central to the research but not yet described in published literature, software must be made available to editors and reviewers. We strongly encourage code deposition in a community repository (e.g. GitHub). See the Nature Portfolio guidelines for submitting code & software for further information.

Data

Policy information about availability of data

All manuscripts must include a data availability statement. This statement should provide the following information, where applicable:

- Accession codes, unique identifiers, or web links for publicly available datasets
- A description of any restrictions on data availability
- For clinical datasets or third party data, please ensure that the statement adheres to our policy

All data reported in this paper were deposited on Zenodo and are available for public download (https://doi.org/10.5281/zenodo.8048351).

Research involving human participants, their data, or biological material

Policy information about studies with human participants or human data. See also policy information about sex, gender (identity/presentation), and sexual orientation and race, ethnicity and racism.

Reporting on sex and gender	Participants were asked to report their gender (male/female/other/prefer not to answer).<br>Study 1: 14 male, 21 female, 1 non-binary<br>Study 2: 17 male, 13 female<br>Study 3: 21 male, 14 female<br>Study 4: 8 male, 13 female<br>Since gender is not a covariate of interest in our studies, we had no gender-related analyses
Reporting on race, ethnicity, or other socially relevant groupings	We do not report on race/ethnicity.
Population characteristics	See below
Recruitment	For the EEG studies, participants were recruited on campus, mostly through the SONA system.<br>For the behavioral studies, participants were recruited on Prolific.
Ethics oversight	Institutional Review Board at the University of California, Berkeley

Note that full information on the approval of the study protocol must also be provided in the manuscript.

Field-specific reporting

Please select the one below that is the best fit for your research. If you are not sure, read the appropriate sections before making your selection.

☐ Life sciences ☒ Behavioural & social sciences ☐ Ecological, evolutionary & environmental sciences

For a reference copy of the document with all sections, see nature.com/documents/nr-reporting-summary-flat.pdf

Behavioural & social sciences study design

All studies must disclose on these points even when the disclosure is negative.

Study description	We had 2 EEG studies and 2 behavioral studies. For all studies the data are quantitative.
Research sample	For both EEG studies, we recruited UC Berkeley undergraduates and graduates through the campus SONA system.<br><br>For the 2 behavioral studies, we recruited participants on Prolific from the UK and the US.
Sampling strategy	For both EEG studies, we aimed to get a final sample of 30-35 participants, which is the norm in the field. Knowing that some datasets might be unusable because they would contain too few artifact-free trials (see below), we recruited 40 participants.<br><br>The first behavioral study uses a novel paradigm, and we had no way to determine ahead of time what would be a good sample size. We decided to recruit 50 participants. 16 failed three times on the comprehension test and did not play the game, and we excluded 5 more participants (see below). Our final sample was thus composed of 30 participants.<br>Since the second behavioral study is a replication of the study above (with a change of parameter), we aimed for a similar sample size. We initially recruited 61 participants, and ended up with a final sample of 21 (see below).
Data collection	EEG studies were conducted in the lab, with the EEG Biosemi setup. Participants sat in front of a computer and played a slot machine game using the keyboard. An experimenter read the instructions aloud and stayed with the participants during the practice trials. Participants were left alone in the EEG booth during the experiment itself.<br>The behavioral studies ran online. Participants used their own computer and completed the study by themselves.
Timing	EEG Study 1 was conducted from May to September 2021.<br>EEG Study 2 was conducted from October 2021 to September 2022<br>Behav Study 1 was conducted in January 2022<br>Behav Study 2 was conducted in January 2023
Data exclusions	Study 1 (EEG): The experiment was conducted on 42 participants. Data from 6 participants were excluded because too few artifact-free trials were available (<80% of all trials).<br>Study 3 (EEG): Same as Study 1. The experiment was conducted on 41 participants. Data from 6 participants were excluded because too few artifact-free trials were available.<br>Study 2 (Behavioral, online): 51 participants were recruited online. 16 participants failed three times on the comprehension test and did not play the game. The main task of participants was to switch back and forth between two choice options (a sure amount of money and a gamble) during the trial, as they were continuously provided with updates regarding the chances of winning associated

	with the gamble. We excluded 5 participants who did not switch at all in 90% or more of the trials, including in the 750ms time window when the outcome of the gamble was already known.<br>Study 4: Same as Study 2. 61 participants were recruited online. 32 participants failed three times on the comprehension test and did not play the game. We excluded 8 participants who did not switch at all in 90% or more of the trials.
Non-participation	No participants dropped out.
Randomization	Participants were not allocated into experimental groups as all experiments used a within-subject design.

Reporting for specific materials, systems and methods

We require information from authors about some types of materials, experimental systems and methods used in many studies. Here, indicate whether each material, system or method listed is relevant to your study. If you are not sure if a list item applies to your research, read the appropriate section before selecting a response.

Materials & experimental systems

n/a	Involved in the study
☒	☐ Antibodies
☒	☐ Eukaryotic cell lines
☒	☐ Palaeontology and archaeology
☒	☐ Animals and other organisms
☒	☐ Clinical data
☒	☐ Dual use research of concern
☒	☐ Plants

Methods

n/a	Involved in the study
☒	☐ ChIP-seq
☒	☐ Flow cytometry
☒	☐ MRI-based neuroimaging
